# Ten-Year Voriconazole Susceptibility Trends Among 1482 Clinical *Aspergillus fumigatus* Isolates: A Compositionally Controlled Multicenter Analysis of the VIVLI/SENTRY Platform (2010–2020)

**DOI:** 10.3390/jof12090699

**Published:** 2026-09-18

**Authors:** Jose W. Lopez, Alicia A. Segura-Grados, Emily Olascoaga-Mori, Harold J. Rodríguez-Madueño

**Affiliations:** 1 Facultad de Ciencias de la Salud, Universidad Científica del Sur, Lima 15067, Peru; 100065822@cientifica.edu.pe (A.A.S.-G.); 100066174@cientifica.edu.pe (E.O.-M.); 100066896@cientifica.edu.pe (H.J.R.-M.); 2 Instituto Nacional de Salud del Niño San Borja, Lima 15037, Peru

**Keywords:** *Aspergillus fumigatus*, voriconazole, azole resistance, antifungal susceptibility, SENTRY antifungal surveillance program, VIVLI, CLSI breakpoints, multicenter surveillance

## Abstract

Azole resistance in *Aspergillus fumigatus* is an emerging threat to invasive and chronic aspergillosis management, yet multicenter trend analyses rarely verify whether the composition of surveillance data remained stable over time. We performed a longitudinal, secondary-data analysis of 2111 *Aspergillus* isolates registered in the VIVLI AMR platform (ATLAS Antifungals, SENTRY Program) between 2010 and 2020. Because Clinical and Laboratory Standards Institute (CLSI) voriconazole breakpoints are established only for *A. fumigatus* in this dataset, the susceptibility-trend analysis was restricted to *A. fumigatus* isolates with a valid interpretation (n = 1482/1483). *A. fumigatus* accounted for 70.3% of all *Aspergillus* isolates; most originated from Europe (43.9%) and North America (35.2%). Region and clinical specialty differed significantly across the three study periods (both *p* < 0.0001), while species, age, sex and specimen-source distributions remained stable (*p* > 0.10). Voriconazole susceptibility in *A. fumigatus* declined from 97.9% (2010) to 90.4% (2020), with a significant linear downward trend (Cochran–Armitage Z = −3.03, *p* = 0.002). This decline was statistically robust, but the concurrent shift in participating regions and specialties indicates that compositional changes in surveillance sampling should be considered alongside biological resistance emergence when interpreting multicenter antifungal surveillance trends.

## 1. Introduction

Invasive and chronic pulmonary aspergillosis remain major causes of morbidity and mortality among immunocompromised and critically ill patients, and azole antifungals—itraconazole, voriconazole, posaconazole and isavuconazole—constitute the cornerstone of prophylaxis and treatment for *Aspergillus fumigatus* infections [1]. Over the past two decades, azole-resistant *A. fumigatus* has emerged worldwide, driven primarily by mutations in the CYP51A gene (e.g., TR34/L98H, TR46/Y121F/T289A) that arise under both clinical antifungal exposure and environmental selection pressure from agricultural azole fungicides [1,2]. Resistant isolates have now been reported on every inhabited continent, although prevalence and resistance mechanisms vary substantially by region, with the highest documented rates in Western Europe and comparatively limited surveillance data from Latin America, Asia and Oceania [1,2,3].

Multicenter surveillance platforms are essential to characterize these trends beyond single-center reports, which are subject to local selection bias. The SENTRY Antimicrobial Surveillance Program, accessible through the VIVLI global data-sharing platform, has monitored antimicrobial and antifungal susceptibility across sentinel medical centers worldwide since 1997 using centralized, standardized reference testing, providing one of the largest secondary-analysis-ready isolate collections available [4].

However, temporal-trend analyses derived from multicenter surveillance data face an underappreciated methodological challenge: because participating centers, geographic regions and clinical specialties are not fixed across years, an apparent change in susceptibility may partly reflect a change in the composition of contributing sites rather than a true shift in the pathogen’s biology. This concern is particularly relevant for decade-long datasets such as ATLAS/SENTRY, in which the network of contributing laboratories evolves over time. To our knowledge, few reports have explicitly evaluated whether the demographic and clinical composition of *Aspergillus* surveillance data remained stable before interpreting susceptibility trends.

The aim of this study was therefore twofold: first, to characterize the evolution of voriconazole susceptibility in *A. fumigatus* isolates from the VIVLI/SENTRY ATLAS Antifungals dataset between 2010 and 2020; and second, to assess whether the demographic, geographic and clinical-specialty composition of the sampled isolates remained stable across the study period, in order to contextualize the observed susceptibility trend.

## 2. Materials and Methods

### 2.1. Study Design and Data Source

This was a longitudinal, secondary-data analysis of isolates registered in the VIVLI AMR data-sharing platform (Burlington, MA, USA) [5], ATLAS Antifungals dataset, generated through the SENTRY Antifungal Surveillance Program [4]. Isolates were collected from participating medical centers worldwide and centrally identified and tested for antifungal susceptibility using Clinical and Laboratory Standards Institute (CLSI) broth microdilution methodology, with categorical interpretation (Susceptible/Intermediate/Resistant; SIR) applied using CLSI epidemiological cutoff values/breakpoints where established [6].

### 2.2. Study Population and Eligibility Criteria

The inclusion/exclusion process is summarized in Table 1. Of 19,596 antifungal isolates registered in the source dataset (2010–2020, all genera), 2111 had the value “*Aspergillus*” in the source variable labelled Family. In this export, that value functioned as a genus-level taxonomic filter; it does not indicate that *Aspergillus* is a biological family. These 2111 isolates constituted the analytic population for the general demographic and clinical description (Aim 1). No isolates were excluded because of missing demographic data (sex, age); these were retained and reported under an explicit “Unknown/Missing” category to preserve the full denominator and avoid selection bias from complete-case restriction.

For the voriconazole susceptibility-trend analysis (Aim 2), the population was further restricted to *A. fumigatus* isolates (n = 1483), because CLSI voriconazole interpretive breakpoints were available almost exclusively for this species in the source dataset: the CLSI categorical interpretation was missing in 100% of non-fumigatus *Aspergillus* isolates versus 0.07% (1/1483) of *A. fumigatus* isolates. Isolates with a valid (non-missing) voriconazole CLSI interpretation (n = 1482) constituted the final analytic sample for the trend analysis.

### 2.3. Variable Definitions

The following variables and recoding rules were defined explicitly and documented for reproducibility:

• Sex: as recorded in the Gender field (Male, Female, Unknown/Missing).
• Age group: recoded from the original four-level classification into 0–17, 18–60 (merging 18–30 and 31–60), 61+ years, and Unknown/Missing.
• Study period: three intervals (2010–2013, 2014–2017, 2018–2020), chosen to retain adequate cell counts for compositional testing.
• Species: *A. fumigatus*, *A. flavus*, *A. niger*, *A. terreus*, and other *Aspergillus* spp.; species-complex designations were merged with the corresponding species.
• Specimen source: grouped as respiratory tract (sputum, bronchoalveolar lavage/wash, tracheal aspirate, lower/upper respiratory tract, invasive pulmonary) versus non-respiratory (all other sites, including tissue, sterile fluids, blood, cerebrospinal fluid and bone/joint). A dichotomous grouping was adopted because sterile-site categories individually contained fewer than 25 isolates, producing expected cell counts below 5.
• Geographic region: countries mapped to five regions (North America, South America, Europe, Asia, Oceania); Turkey was classified as Europe, consistent with common SENTRY/ATLAS reporting conventions.
• Clinical specialty: taken from the Specialty field of the source dataset (the hospital service submitting the isolate) and grouped into Internal Medicine; Cardiothoracic/Pulmonary; Critical Care (intensive care unit); Hematology/Oncology & Transplant; Ambulatory/Outpatient; Surgery (general, neuro-, orthopedic, trauma, burn, urological and obstetric/gynecological); Pediatrics/Neonatology; Other; and Unknown/Missing. Grouping was required because the raw field contains 29 non-missing levels, of which 55% of cells had expected counts below 5, and because it contains duplicate levels differing only in spelling (“Ear, Nose, Throat”/“Ear, Nose, Throat (Otolaryngology)”; “Long Term Care”/“Long-Term Care”), which were merged.
• Voriconazole interpretation: the CLSI SIR category recorded in the source dataset; susceptibility was analyzed as the binary proportion Susceptible versus non-susceptible (Intermediate or Resistant).

### 2.4. Statistical Analysis

Categorical variables were summarized as frequencies and percentages. To assess whether the demographic and clinical composition of the sample was stable over time, chi-square tests of independence were used to test the association between each compositional variable (sex, age group, species, specimen-source group, region, clinical specialty) and the three-year study period, excluding the Unknown/Missing category from each test. For the primary outcome—the temporal trend in voriconazole susceptibility among *A. fumigatus* isolates—the annual percentage of susceptible isolates was calculated, and a Cochran–Armitage trend test was used to formally test for a linear trend in the proportion of susceptible isolates across years; this test is statistically more appropriate than an omnibus chi-square test for an ordinal exposure (calendar year) with a binary outcome. Ninety-five percent confidence intervals for each annual point estimate of susceptibility were calculated using the Wilson score method, which performs more reliably than the normal approximation for the smaller yearly sample sizes observed early in the series (e.g., n = 48 in 2010–2011). A conventional chi-square test of independence (year × three-category CLSI interpretation) was also reported for comparability with prior surveillance literature. Statistical significance was set at α = 0.05. Analyses were performed in R version 4.5.3 (R Core Team, Vienna, Austria) using the data.table package (version 1.18.4) following a fully scripted, modular pipeline; the analysis script is available as Supplementary Material File S1 to allow independent verification of every reported figure.

### 2.5. Ethical Considerations

This study used a de-identified, publicly available secondary surveillance dataset accessed with authorization from the VIVLI Data Access Committee; no new data involving human or animal subjects were collected by the authors, and no additional ethical approval or informed consent was required.

### 2.6. Use of Generative Artificial Intelligence

During the preparation of this manuscript, the authors used Claude (Anthropic) to assist with data-cleaning code review, descriptive and inferential statistical computation, and drafting of text based on author-provided results and study parameters. The authors reviewed, verified against the source dataset, and edited all AI-assisted output, and take full responsibility for the content of this publication.

## 3. Results

### 3.1. Study Population

Of 19,596 antifungal surveillance isolates registered in the ATLAS Antifungals dataset (2010–2020), 2111 (10.8%) belonged to the genus *Aspergillus* and constituted the analytic population (Table 1 and Table 2). Patients aged over 60 years accounted for the largest share of isolates (39.2%), followed by those aged 18–60 years (37.1%); age was unrecorded for 14.5% of isolates. Isolates were more frequent among male patients (49.1% vs. 39.0% female; 11.9% unrecorded). By study period, 21.3% of isolates were collected in 2010–2013, 42.1% in 2014–2017, and 36.6% in 2018–2020. *A. fumigatus* was the predominant species (70.3%), followed by *A. flavus* (10.2%), *A. niger* (10.1%) and *A. terreus* (3.8%). The remaining 120 isolates (5.7%) were pooled as other *Aspergillus* spp. for the primary summary; their species-level composition is detailed below. Most isolates originated from Europe (43.9%) and North America (35.2%), with smaller contributions from Oceania (9.3%), Asia (7.1%) and South America (4.5%).

**Table 2:** Demographic and clinical characteristics of *Aspergillus* isolates, 2010–2020 (n = 2111).

Variable	Category	n	%
Age group	0–17 years	194	9.2
	18–60 years	783	37.1
	61+ years	827	39.2
	Unknown/Missing	307	14.5
Sex	Male	1036	49.1
	Female	823	39.0
	Unknown/Missing	252	11.9
Study period	2010–2013	449	21.3
	2014–2017	889	42.1
	2018–2020	773	36.6
Species	*A. fumigatus*	1483	70.3
	*A. flavus*	215	10.2
	*A. niger*	213	10.1
	*A. terreus*	80	3.8
	Other *Aspergillus* spp.	120	5.7
Region	Europe	927	43.9
	North America	743	35.2
	Oceania	196	9.3
	Asia	150	7.1
	South America	95	4.5
Specimen source	Respiratory tract	1561	74.0
	Non-respiratory	550	26.0
Clinical specialty	Internal Medicine	433	20.5
	Unknown/Missing	363	17.2
	Cardiothoracic/Pulmonary	341	16.2
	Critical Care	250	11.8
	Other	202	9.6
	Hematology/Oncology & Transplant	164	7.8
	Ambulatory/Outpatient	149	7.1
	Surgery	114	5.4
	Pediatrics/Neonatology	95	4.5

The other *Aspergillus spp.* comprised *A. nidulans* (n = 39), *A. versicolor* (n = 10), *A. nidulans species complex* (n = 9), *A. sydowii*, *A. tubingensis*, *A. ustus species complex*, *A. ustus* and *A. lentulus* (n = 7 each); a full listing is given in Supplementary Table S1. Notably, this group includes cryptic species with intrinsically reduced azole susceptibility (*A. lentulus, A. thermomutatus, A. ustus*), none of which could be evaluated here because CLSI voriconazole breakpoints were unavailable for all non-fumigatus isolates.

Percentages calculated over n = 2111; Unknown/Missing retained to preserve the full denominator (Section 2.2). Other Aspergillus spp. comprised 23 taxa (Supplementary Table S1). Specialty and specimen-source groupings are defined in Section 2.3.

### 3.2. Compositional Stability Across the Study Period

Chi-square tests of association between each compositional variable and the three-year study period are reported in Table 3. Clinical specialty comprised 29 non-missing source categories, retained and analyzed without collapsing. Region and clinical specialty differed significantly across periods (both *p* < 0.0001), indicating that the geographic and clinical-setting mix of participating sites changed over the study decade. In contrast, sex, age group, species and specimen-source distributions did not differ significantly across periods (all *p* > 0.10), indicating that these characteristics of the sampled isolates remained comparatively stable over time.

**Table 3:** Association between compositional variables and study period (chi-square test of independence).

Variable	χ^2^	df	*p*-Value
Sex	0.76	2	0.682
Age group	7.73	4	0.102
Species	10.45	8	0.235
Specimen source	2.71	4	0.607
Region	124.90	8	<0.0001
Clinical specialty	235.36	56	<0.0001

Unknown/Missing category excluded from each test. Clinical specialty comprised 29 non-missing categories analyzed as recorded. df = degrees of freedom.

The 29 non-missing clinical-specialty categories were Internal Medicine; Cardiothoracic/Pulmonary; Intensive Care Unit; Ambulatory/Outpatient; Hematology/Oncology; Pediatrics/Neonate; Surgery; Infectious Disease; Ear, Nose, Throat; Neurology; Transplant; General/GI; Geriatrics; Neurosurgery; Family Practice; Ear, Nose, Throat (Otolaryngology); Emergency; Renal; Orthopedics; Obstetrics/Gynecology; Burn; Ophthalmology; Rehabilitation; Trauma; Dialysis; Dermatology; Long Term Care; Long-Term Care; and Urology. The two ear, nose and throat labels and the two long-term-care labels were retained as distinct source categories, as recorded in the dataset.

### 3.3. Evolution of Voriconazole Susceptibility in A. fumigatus

**Figure 1:**
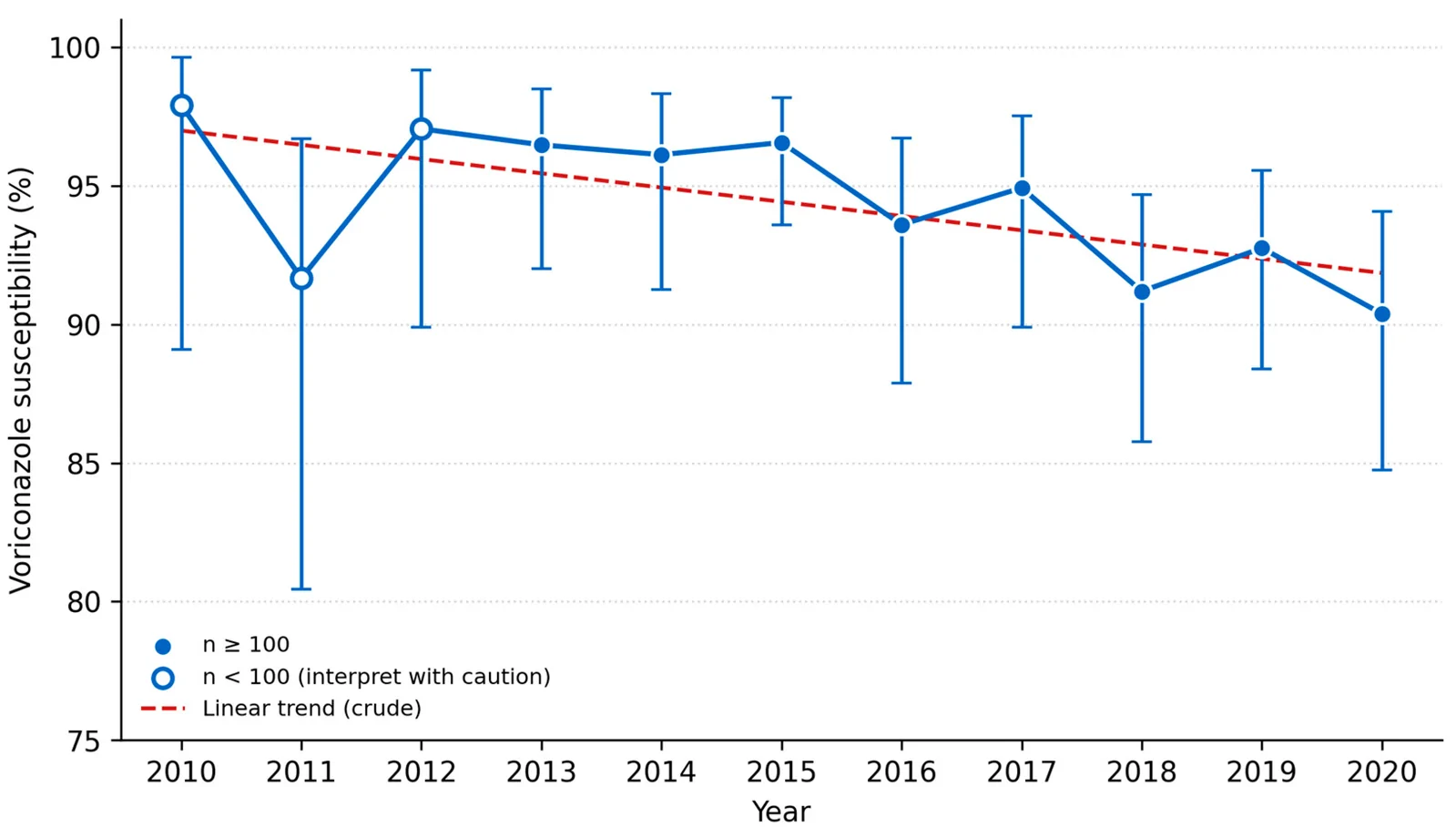
Crude annual percentage of voriconazole-susceptible A. fumigatus isolates, 2010–2020 (n = 1482). Error bars are 95% Wilson score confidence intervals. Open markers denote years with fewer than 100 isolates, whose point estimates should be interpreted with caution. Dashed line: ordinary least-squares linear trend.

Among 1482 *A. fumigatus* isolates with a valid CLSI interpretation, voriconazole susceptibility declined from 97.9% in 2010 (n = 48) to 90.4% in 2020 (n = 156), with the most pronounced decrease occurring after 2016 (Table 4, Figure 1). No isolates were classified as resistant in 2011 or 2015; however, 4 of 48 isolates (8.3%) in 2011 and 9 of 262 isolates (3.4%) in 2015 were classified as intermediate, accounting for susceptibility below 100% in those years. The omnibus chi-square test of the full year × three-category SIR table was not statistically significant (χ^2^ = 27.84, df = 20, *p* = 0.113), reflecting sparse cell counts for the Intermediate and Resistant categories in individual years. The Cochran–Armitage trend test, which is more appropriate for testing an ordered linear trend in a binary proportion, confirmed a statistically significant declining trend in susceptibility across the study period (Z = −3.03, *p* = 0.002).

**Table 4:** Annual voriconazole susceptibility in *A. fumigatus*, 2010–2020 (n = 1482).

Year	n	n Susceptible	% Susceptible	95% CI (Wilson)	% Resistant
2010	48	47	97.92	89.10–99.63	2.08
2011	48	44	91.67	80.45–96.71	0.00
2012	68	66	97.06	89.90–99.19	1.47
2013	142	137	96.48	92.02–98.49	1.41
2014	129	124	96.12	91.25–98.33	0.78
2015	262	253	96.56	93.60–98.18	0.00
2016	125	117	93.60	87.88–96.72	0.80
2017	138	131	94.93	89.90–97.52	2.17
2018	159	145	91.19	85.76–94.68	3.77
2019	207	192	92.75	88.39–95.56	2.90
2020	156	141	90.38	84.74–94.09	4.49

95% CI: Wilson score confidence interval for the annual proportion susceptible. Cochran–Armitage trend test: Z = −3.03, *p* = 0.002. Global χ^2^ (year × 3-category SIR) = 27.84, df = 20, *p* = 0.113.

### 3.4. Contribution of Changing Country Composition to the Observed Trend

The 28 resistant isolates were not distributed evenly across the network but concentrated in a few countries with high within-country resistance prevalence that entered the dataset after the start of the series. Italy contributed 10 resistant isolates of 71 (14.1%) and provided no isolates before 2015; the United Kingdom contributed 4 of 18 (22.2%) and none before 2019; Belgium contributed 2 of 23 (8.7%) and none before 2018. Together these three countries supplied 16 of the 28 resistant isolates (57%) while being entirely absent from the dataset in 2011, and largely absent throughout the first period. This directly explains the two features the crude series displays: the near-absence of resistant isolates early on, and their accumulation after 2018.

## 4. Discussion

In this multicenter analysis of 1482 *A. fumigatus* isolates from the VIVLI/SENTRY ATLAS Antifungals dataset, voriconazole susceptibility declined significantly between 2010 and 2020, consistent with the worldwide emergence of azole-resistant *A. fumigatus* documented over the past two decades [1,2,7]. The overall proportion of non-susceptible isolates observed here (9.6% by 2020, combining Intermediate and Resistant categories) falls within the wide range reported across prior surveillance studies, which varies considerably depending on region, sampling frame (clinical vs. environmental), the denominator used for resistance calculation, and the surveillance methodology employed [1,8]. Because our dataset aggregates isolates predominantly from Europe (43.9%) and North America (35.2%), these findings most directly complement European and North American surveillance literature, where clinical azole-resistance prevalence has historically ranged from under 1% to over 20% depending on the country and period studied [1,9,10].

The isolated decrease in susceptibility in 2011 should be interpreted cautiously. The annual sample was small (n = 48), the 95% Wilson confidence interval was wide (80.45–96.71%), and all four non-susceptible isolates were classified as intermediate rather than resistant. Likewise, the absence of resistant isolates in 2015 did not indicate complete susceptibility: nine of 262 isolates were intermediate. Given the significant changes in regional and clinical-specialty composition across the broader study periods, year-to-year sampling variation may have contributed to these fluctuations [4,8]; however, the available aggregate data do not permit a definitive year-specific attribution. These observations support emphasizing the overall Cochran–Armitage trend rather than interpreting any single annual point in isolation.

Two features of the early series illustrate the same point at finer grain. The 2011 estimate deviates from its neighbors on the basis of four Intermediate isolates in a sample of 48 drawn from an atypical set of contributors: Brazil supplied 9 of 48 isolates that year (18.8%, against 1.3–5.0% in most other years) and Slovakia supplied 10, having contributed in 2010 and 2011 only and never again. Similarly, the absence of resistant isolates in 2015 coincides with a year in which Italy had only just joined and the United Kingdom and Belgium had not, so no high-prevalence contributor was represented. In a network of this size, single-center entries and exits move annual estimates more than plausible year-on-year changes in fungal biology.

Our results align closely with recent SENTRY program analyses covering overlapping periods. Pfaller et al. evaluated 731 *A. fumigatus* isolates from Europe (n = 449) and North America (n = 282) collected between 2017 and 2021, and found that the overall frequency of azole-resistant non-wild-type (NWT) isolates had risen to approximately 10.8% (10.7% in Europe, 11.0% in North America), approaching the 10% threshold above which expert guidelines recommend reconsidering azole monotherapy for empirical treatment of invasive aspergillosis [8,11]. Notably, that study documented a steady increase in azole-resistant NWT isolates from North America, from 6.0% in 2017 to 29.3% in 2021, while European rates fluctuated between 4.9% and 20.6% without a consistent temporal trend [11]. Our observation of a significant linear decline in voriconazole susceptibility over 2010–2020 is therefore consistent with—and extends backward in time—the emerging picture from SENTRY and other surveillance platforms indicating that azole resistance in *A. fumigatus* is no longer confined to historically high-prevalence regions of Western Europe [1,9,11]. National surveillance programs have reported similarly concerning trajectories: in the Netherlands, azole-resistant *A. fumigatus* increased from 7.6% in 2013 to 14.7% in 2018, with TR34/L98H and TR46/Y121F/T289A mutations accounting for 86% of CYP51A alterations [9], while a prospective multicenter international surveillance study across 22 centers reported an overall resistance prevalence of 3.2% [7].

The clinical significance of this declining susceptibility trend is substantial. Voriconazole resistance in invasive aspergillosis has been associated with an excess overall mortality of 21% at day 42 and 25% at day 90 compared to susceptible infections, and delayed initiation of appropriate antifungal therapy further increases mortality [12]. In hematology patients specifically, voriconazole-resistant invasive aspergillosis has been linked to significantly higher 12-week mortality compared to susceptible infections [13]. A prospective multicenter study of 323 hematology patients across 12 centers found that azole resistance was detectable by PCR in 14% of cases with conclusive resistance testing, and that real-time resistance detection enabling prompt therapy modification was associated with a reduction in treatment failure from a historical 75% to 16.7% [14]. These findings underscore that the susceptibility decline observed in our dataset, while modest in absolute terms, translates into clinically meaningful consequences when extrapolated to the population of patients at risk for invasive aspergillosis. The 9.6% non-susceptibility rate we observed by 2020 approaches the 10% threshold above which current expert opinion recommends reconsidering voriconazole monotherapy as empirical first-line therapy [8, 11].

The mechanisms underlying this global resistance trend are increasingly well characterized. Azole resistance in *A. fumigatus* is driven primarily by mutations in the CYP51A gene, with the tandem-repeat mutations TR34/L98H and TR46/Y121F/T289A being the most prevalent and widely disseminated resistance mechanisms worldwide [1,2,15]. These environmental-type mutations are thought to arise under selection pressure from agricultural azole fungicides rather than clinical antifungal exposure, and their global spread has been documented through population genomic studies showing near-identical genotypes between environmental and clinical resistant isolates, confirming environmental acquisition of resistant infections by humans [15,16]. However, the geographic distribution of resistance mechanisms is heterogeneous: in the SENTRY 2017–2021 analysis, the TR34/L98H alteration was detected exclusively in European isolates (accounting for 72.0% of European NWT isolates), while North American NWT isolates harbored distinct non-environmental point mutations such as I242V (58.3% of North American NWT), suggesting that resistance in North America may arise primarily from drug pressure in the healthcare setting rather than environmental fungicide exposure [11]. This mechanistic dichotomy has important implications for interpreting pooled multicenter trends such as ours: because our dataset aggregates isolates from regions with fundamentally different resistance drivers, the observed susceptibility decline may reflect a composite of environmentally and clinically driven resistance emerging at different rates across contributing regions.

A central methodological contribution of this analysis is the explicit test of compositional stability across the study period. We found that the geographic region and clinical specialty of contributing isolates changed significantly over time, while species, age, sex and specimen-source distributions did not. This asymmetry is informative: it suggests that the declining voriconazole susceptibility trend is unlikely to be a simple artifact of shifting species mix or patient demographics, but it also means that changes in which regions and clinical settings contributed isolates over time cannot be ruled out as partial contributors to the observed trend, given the known geographic heterogeneity in resistance prevalence and mechanisms [1,2,11]. This concern is not merely theoretical. Verweij et al. argued that azole resistance surveillance data are subject to numerous potential biases—including selection bias, referral bias, and denominator-dependent variation—that can substantially affect prevalence estimates and temporal trends [8]. The wide range of reported resistance frequencies (from <1% to >20%) across surveillance studies reflects not only genuine geographic variation but also differences in surveillance design, including whether isolates are screened unselected, drawn from specific patient cohorts, or focused on particular risk groups [8]. Furthermore, Sallam has highlighted a related but distinct confounder in longitudinal resistance surveillance: “breakpoint drift”, whereby revisions to CLSI and EUCAST interpretive breakpoints over time can inflate apparent resistance rates independent of any biological change in the organism, analogous to how redefining diagnostic thresholds in chronic disease classification increases disease prevalence without true changes in pathology [17]. Although CLSI voriconazole breakpoints for *A. fumigatus* remained stable during our study period, this principle reinforces the broader argument that temporal-trend analyses of surveillance data must explicitly account for methodological and compositional changes alongside biological signals. Our finding echoes the broader methodological principle, previously highlighted in relation to *Aspergillus* azole-resistance surveillance in Latin America, that regional and site-level variability limits direct extrapolation of pooled multicenter trends to any single setting [3], and argues for routinely reporting compositional-stability diagnostics alongside temporal-trend statistics in future ATLAS/SENTRY analyses.

The geographic composition of our dataset also warrants discussion in the context of global surveillance gaps. Europe and North America together contributed 79.1% of isolates, while South America accounted for only 4.5%, Asia 7.1%, and Oceania 9.3%. This overrepresentation of European and North American centers is a well-recognized feature of the SENTRY program and similar voluntary surveillance networks [4,8], and it means that our susceptibility trend primarily reflects the epidemiology of regions with established resistance surveillance infrastructure. The situation in Latin America remains poorly characterized, despite evidence that azole-resistant *A. fumigatus* is present in the region. Bustamante et al. reported the first clinical azole resistance data from Peru, finding that 2% (3/143) of *A. fumigatus* clinical isolates from Lima were resistant to itraconazole, including one isolate harboring the TR34/L98H mutation from an azole-naive patient with chronic pulmonary aspergillosis—providing the first clinical evidence of environmentally acquired resistance in South America [18]. Environmental surveillance studies have documented triazole-resistant *A. fumigatus* in Mexico, Peru, and Paraguay, with environmental prevalence of 6.9% in Mexico and 9.8% in Peru [19]. These findings indicate that resistance is present in Latin America at levels that may be clinically significant, yet the region remains severely underrepresented in multicenter surveillance platforms. The significant shift in regional composition we observed over the study period (Table 3) thus has implications for generalizability: if South American or Asian participation were to increase in future surveillance cycles, pooled susceptibility estimates could shift in ways that reflect changing sampling composition rather than biological trends.

The public health importance of continued azole resistance surveillance is underscored by the inclusion of *A. fumigatus* in the World Health Organization’s first fungal priority pathogens list, published in 2022, where it was designated among the four critical-priority fungal pathogens [20,21]. This designation reflects the high mortality associated with invasive aspergillosis (estimated at 30–50% in immunocompromised patients), the limited number of antifungal drug classes available for treatment, and the growing threat of azole resistance [10,20,21]. The WHO report and accompanying systematic review emphasized that surveillance for azole resistance remains inadequate in many regions and that strengthened global monitoring is essential to inform treatment guidelines and public health policy [20,21]. Our analysis, by demonstrating a statistically significant susceptibility decline over a decade while simultaneously flagging the compositional instability of the underlying surveillance data, illustrates both the value and the limitations of existing multicenter surveillance platforms for meeting this public health need.

From a therapeutic perspective, the declining voriconazole susceptibility we observed reinforces the importance of antifungal stewardship and the development of alternative treatment strategies for azole-resistant aspergillosis. When local resistance rates exceed 10%, expert guidelines recommend reconsidering voriconazole monotherapy in favor of alternative agents or combination regimens [8,11]. Isavuconazole has demonstrated in vitro activity comparable to voriconazole against *A. fumigatus*, with WT rates of 92.7% in Europe and 94.0% in North America in the SENTRY 2017–2021 analysis, and remains active against a subset of azole-resistant NWT isolates [11]. Combination therapy with isavuconazole and echinocandins has shown synergistic in vitro activity against both azole-susceptible and some azole-resistant isolates, though this benefit may be limited for isolates harboring the TR46/Y121F/T289A mutation, which confers high-level isavuconazole resistance [22]. Liposomal amphotericin B remains an alternative for confirmed azole-resistant disease, though its use is limited by nephrotoxicity [23]. These therapeutic considerations highlight that the susceptibility trends documented in surveillance datasets like ATLAS/SENTRY have direct implications for empirical treatment decisions, particularly in regions approaching or exceeding the 10% resistance threshold.

This study has several limitations. First, the dataset does not include a patient identifier, precluding deduplication of isolates that may originate from the same patient across sampling events, which could inflate effective sample size in centers with intensive longitudinal sampling. Second, no molecular resistance-mechanism data (e.g., CYP51A genotyping) were available, so we cannot distinguish environmentally acquired resistance (TR34/L98H, TR46/Y121F/T289A) from resistance selected under clinical azole exposure, nor can we determine whether the observed susceptibility decline reflects the spread of environmental-type resistance, the emergence of clinical point mutations, or a combination of both—as the SENTRY 2017–2021 data suggest that these mechanisms differ by region [11]. Third, CLSI voriconazole breakpoints were available only for *A. fumigatus*, excluding cryptic species with intrinsically reduced azole susceptibility (*A. lentulus, A. thermomutatus, A. ustus*) that were present in the dataset; extension of epidemiological cutoff values to these species would materially improve future surveillance. Fourth, as with any secondary analysis of a voluntary surveillance network, participation is not randomly distributed geographically, and the observed regional shift over time (Table 3) reinforces the need for cautious interpretation of pooled trends; the significant change in clinical specialty composition further suggests that the case mix of contributing sites evolved, which could influence susceptibility estimates if resistance prevalence differs by clinical setting [8]. Fifth, our analysis captures only phenotypic susceptibility categories (S/I/R) without MIC values, which limits the ability to detect more subtle shifts in the MIC distribution that may precede categorical resistance. Finally, the absence of patient-level clinical data precludes assessment of whether resistant isolates were associated with treatment failure or mortality, limiting the clinical interpretability of the observed trend.

Despite these limitations, this analysis makes a distinct contribution to the *Aspergillus* antifungal surveillance literature by explicitly testing and reporting the compositional stability of the surveillance sample alongside the susceptibility trend. Future ATLAS/SENTRY analyses and similar multicenter surveillance studies should routinely incorporate compositional-stability diagnostics—testing whether the geographic, clinical, and demographic composition of contributing isolates remains stable across the study period—as a standard component of temporal-trend interpretation. Additionally, integrating molecular resistance-mechanism data into surveillance platforms would enable distinction between environmentally and clinically driven resistance, providing more actionable information for treatment guidelines. Expanding surveillance participation in underrepresented regions, particularly Latin America, Africa, and Asia, where resistance data remain scarce [1,3,18,19], is essential to build a truly global picture of antifungal resistance epidemiology.

## 5. Conclusions

Voriconazole susceptibility among *A. fumigatus* isolates in the VIVLI/SENTRY ATLAS Antifungals dataset declined significantly between 2010 and 2020. However, the concurrent, statistically significant shift in the geographic and clinical-specialty composition of contributing isolates indicates that compositional changes in surveillance sampling should be explicitly assessed and reported alongside biological resistance trends in multicenter antifungal surveillance analyses. Continued, compositionally aware surveillance and antifungal stewardship remain warranted to monitor the evolving epidemiology of azole resistance in *A. fumigatus*.

## Figures and Tables

**Table 1:** Inclusion/exclusion flow and analytic populations.

Step	Criterion	n	Purpose
N_0_	Raw ATLAS Antifungals export (VIVLI/SENTRY), 2010–2020, all genera	19,596	Source dataset
N_1_	Source field Family = “*Aspergillus*” (genus-level filter)	2111	Descriptive population (Table 2) and association between variables and years of study period (Table 3)
N_2_	Species = *A. fumigatus* (only species with CLSI voriconazole breakpoints)	1483	Outcome-eligible species
N_3_	Valid (non-missing) voriconazole CLSI interpretation	1482	Trend-analysis population (Figure 1, Table 4)

Family is the genus-level organism grouping field of the ATLAS/SENTRY data dictionary; it does not denote the taxonomic family *Aspergillaceae.*

## Data Availability

Restrictions apply to the availability of these data. Data were obtained from the VIVLI AMR Platform (ATLAS Antifungals dataset) and are available at https://amr.vivli.org with the permission of, and upon request and approval by, the VIVLI Data Access Committee. Data accessed on 22 December 2023.

## Supplementary Materials

The following supporting information can be downloaded at: https://www.mdpi.com/article/10.3390/jof12090699/s1, File S1: VIVLI_Aspergillus_voriconazol_analisis. R—fully commented, reproducible analysis script. Table S1: Full species listing of the 120 “other *Aspergillus* spp.” isolates.

## Abbreviations

The following abbreviations are used in this manuscript:

CLSI	Clinical and Laboratory Standards Institute
SIR	Susceptible/Intermediate/Resistant
