# Supplementary material for: Ten-Year Voriconazole Susceptibility Trends Among 1482 Clinical Aspergillus fumigatus Isolates: A Compositionally Controlled Multicenter Analysis of the VIVLI/SENTRY Platform (2010–2020)
Source: J Fungi (Basel). 2026 Sep 18;12(9):699. doi: 10.3390/jof12090699 (PMC13608095; doi:10.3390/jof12090699)
Supplement: Supplementary file 1 [file jof-12-00699-s001.zip › Supplementary_Table_S1_species_listing.pdf]

Supplementary Material

Table S1. Full species listing of the 120 "other Aspergillus spp." isolates

Supplementary to: Ten-Year Voriconazole Susceptibility Trends among 1,482 Clinical *Aspergillus fumigatus* Isolates: A Compositionally Controlled Multicenter Analysis of the VIVLI/SENTRY Platform (2010–2020).

Species (as recorded in ATLAS)	n	% of all Aspergillus (N = 2,111)
Aspergillus nidulans	39	1.85
Aspergillus versicolor	10	0.47
Aspergillus nidulans species complex	9	0.43
Aspergillus ustus species complex	7	0.33
Aspergillus sydowii	7	0.33
Aspergillus tubingensis	7	0.33
Aspergillus ustus	7	0.33
Aspergillus lentulus	7	0.33
Aspergillus parasiticus	4	0.19
Aspergillus alabamensis	3	0.14
Aspergillus tamarii	3	0.14
Aspergillus unguis	3	0.14
Aspergillus nomius	2	0.09
Aspergillus thermomutatus	2	0.09
Aspergillus sclerotiorum	2	0.09
Aspergillus foetidus	1	0.05
Aspergillus clavatus	1	0.05
Aspergillus versicolor species complex	1	0.05
Aspergillus ochraceus species complex	1	0.05
Aspergillus hiratsukae	1	0.05
Aspergillus aculeatus	1	0.05
Aspergillus piperis	1	0.05
Aspergillus hortai	1	0.05
**Total**	**120**	**5.69**

Species-complex designations are reported as recorded in the source dataset. The CLSI voriconazole categorical interpretation was missing for 100% of these isolates, so none contributed to the susceptibility analyses. Species with intrinsically reduced azole susceptibility present in this group include *A. lentulus, A. thermomutatus* and *A. ustus.*
